# Metabolic health phenotype better predicts subclinical atherosclerosis than body mass index-based obesity phenotype in the non-alcoholic fatty liver disease population

**DOI:** 10.3389/fnut.2023.1104859

**Published:** 2023-09-19

**Authors:** Yaqin Wang, Ting Yuan, Shuwen Deng, Xiaoling Zhu, Yuling Deng, Xuelian Liu, Lei Liu, Changfa Wang

**Affiliations:** ^1^Health Management Center, The Third Xiangya Hospital, Central South University, Changsha, China; ^2^Department of General Surgery, The Third Xiangya Hospital, Central South University, Changsha, China

**Keywords:** cross-sectional study, metabolic status, obesity phenotype, subclinical atherosclerosis, non-alcoholic fatty liver disease

## Abstract

**Background:**

Non-alcoholic fatty liver disease (NAFLD), especially lean NAFLD is associated with an increased risk of atherosclerotic cardiovascular disease (CVD). It is not currently known which clinical phenotypes of NAFLD contribute most to individual subclinical atherosclerosis risk. We examined the relationship between body mass index (BMI), the metabolically healthy status, and subclinical atherosclerosis in the NAFLD population.

**Methods:**

Data from asymptomatic NAFLD subjects who participated in a routine health check-up examination were collected. Participants were stratified by BMI (cutoff values: 24.0–27.9 kg/m^2^ for overweight and ≥28.0 kg/m^2^ for obesity) and metabolic status, which was defined by Adult Treatment Panel III criteria. Subclinical atherosclerosis was evaluated by brachial-ankle pulse wave velocity (baPWV) in 27,738 participants and by carotid plaque in 14,323 participants.

**Results:**

Within each BMI strata, metabolically unhealthy subjects had a significantly higher prevalence of subclinical atherosclerosis than metabolically healthy subjects, whereas fewer differences were observed across subjects within the same metabolic category. When BMI and metabolic status were assessed together, a metabolically unhealthy status was the main contributor to the association of clinical phenotypes with the subclinical atherosclerosis burden (all *p* < 0.001). When BMI and metabolic abnormalities were assessed separately, the incidence of subclinical disease did not increase across BMI categories; however, it increased with an increase in the number of metabolic abnormalities (0, 1, 2 and ≥3).

**Conclusion:**

A metabolically healthy status in NAFLD patients was closely correlated with subclinical atherosclerosis, beyond that of the BMI-based obesity phenotype. The application of metabolic phenotyping strategies could enable more precise classification in evaluating cardiovascular risk in NAFLD.

## Graphical abstract

**Graphical Abstract:**
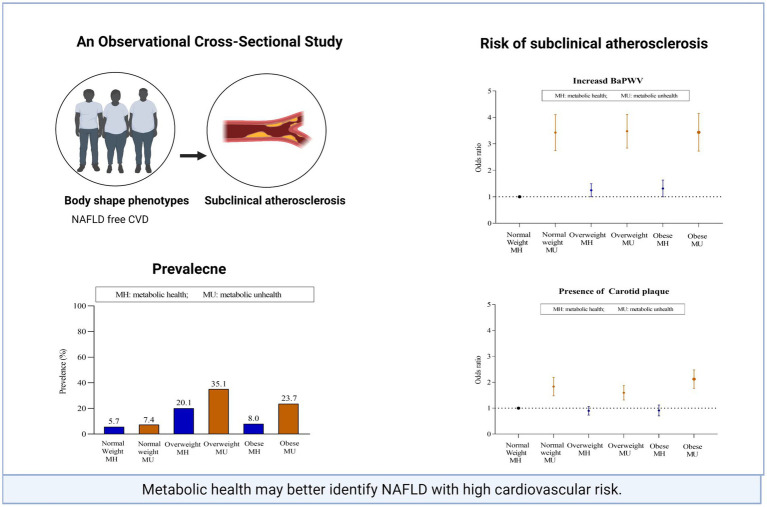

## Introduction

Non-alcoholic fatty liver disease (NAFLD) is currently the most common hepatic disease and a highly heterogeneous metabolic disorder (1, 2). The prognosis of NAFLD is not as benign as thought and confers substantial increases in morbidity and mortality in those individuals who are affected. Evolving data from meta-analyses and cohort studies support the notion that the most common cause of death in the NAFLD population is cardiovascular disease (CVD), followed by extrahepatic malignancies and liver-related complications (3–5).

Although NAFLD is particularly common among subjects with obesity, it is increasingly being identified in lean individuals. The prevalence of lean NAFLD in the NAFLD population ranges from 10% to 20%, with the highest prevalence seen in Asian people (4). In addition, NAFLD interacts with the regulation of multiple metabolic pathways and is bidirectionally linked with components of metabolic syndrome (MetS). In 2020, a panel of international experts proposed that the nomenclature be changed from NAFLD to metabolic dysfunction-associated fatty liver disease (MAFLD) (6). However, the relationship between BMI and metabolic abnormalities is not generally uniform in the NAFLD population (1). There is a subgroup of individuals with obesity who are resistant to metabolic abnormalities, while there is another subset of subjects with normal weight who are prone to metabolic disturbances.

Beyond the association of NAFLD with CVD events, substantial epidemiological evidence links it to subclinical atherosclerosis, including carotid artery intima-media thickness (CIMT), carotid plaque, brachial-ankle pulse wave velocity (baPWV), coronary artery calcification (CAC) and brachial arterial flow-mediated dilation (FMD) (7, 8). However, no studies have investigated the impact of body size and shape combined with metabolic status on cardiovascular risk in the NAFLD population. The underlying relationship between obesity, poor metabolic health and subclinical atherosclerosis remains poorly understood. Awareness of the association is important for clinical practice and could provide proper risk stratification for early lifestyle intervention, which includes a healthy dietary composition in fruits and vegetables, legumes, whole grains, a minimal intake of trans-fats, ultra-processed food, red meat, sugar-sweetened beverages and regular physical activity, and so on improve long-term clinical outcomes (9).

In view of the aforementioned gaps, we examined the associations among the BMI-based obesity phenotype, metabolic health phenotype and subclinical CVD burden, including increased arterial wall stiffness and the presence of carotid plaque, in a large sample of asymptomatic NAFLD subjects without known cardiovascular disease.

## Methods

### Study design and population

**Figure 1:**
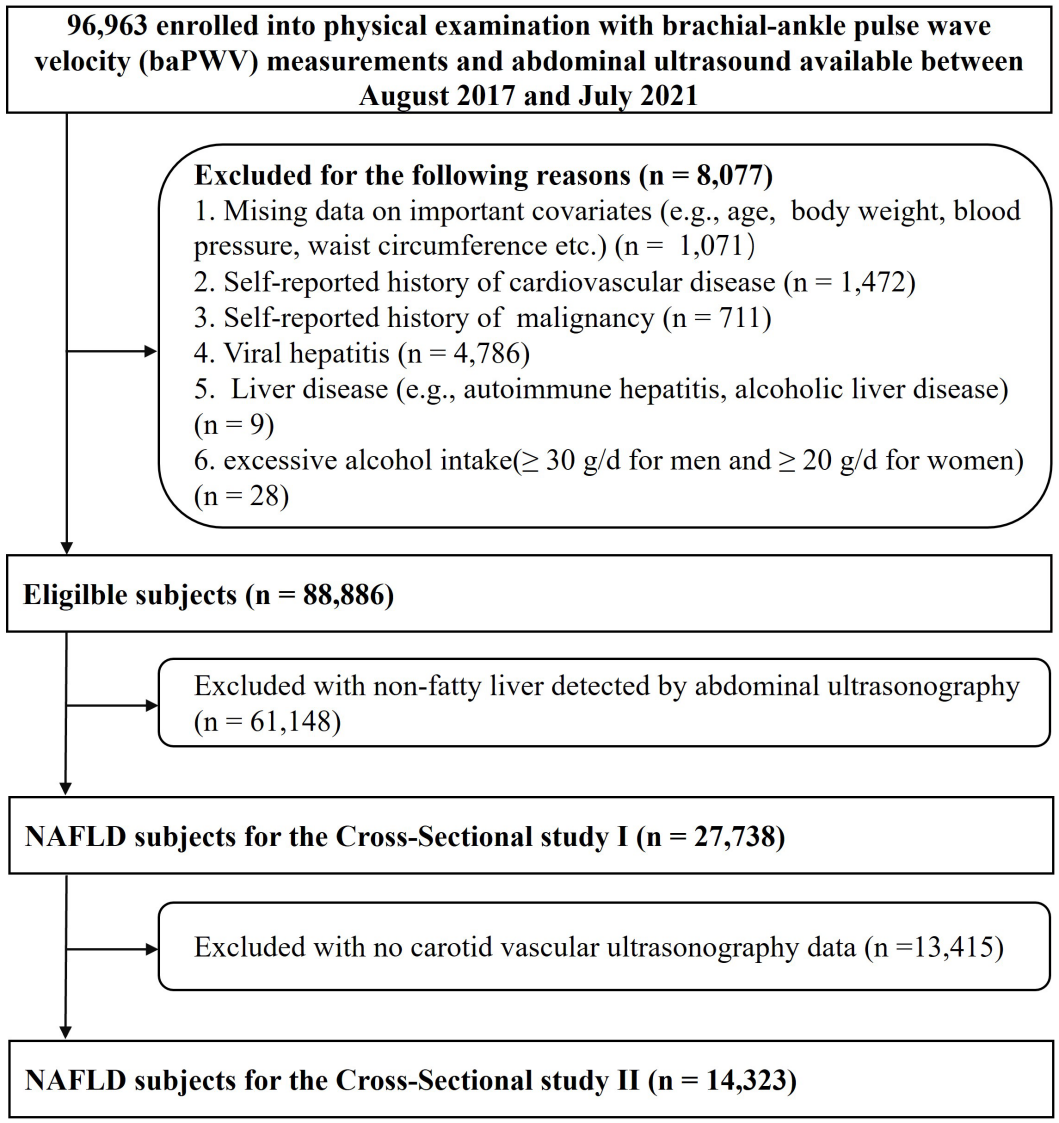
Flow chart of participant selection.

We identified 96,963 adults aged 18–90 years who underwent routine health examinations at the Third Xiangya Hospital of Central South University in Changsha between August 2017 and July 2021. The population consisted of a mix of urban and rural residents. After excluding participants with incomplete information, prior cardiovascular disease, and malignancy and those not meeting the definition of NAFLD, 27,738 NAFLD subjects remained for the cross-sectional study 1 analyses of the risk associated with arterial stiffness. We further excluded participants with no carotid vascular ultrasonography, leaving a final sample of 14,323 NAFLD participants for the cross-sectional study 2 analyses of the risk associated with carotid plaque (Figure 1). Informed consent and the protocol of the overall physical examination were reviewed and approved by the institutional review board at the Third Xiangya Hospital (No. 2018-S393).

### Clinical characteristics

All participants completed a computerized National Physical Examination Questionnaire (10, 11). Personal details (demographic characteristics, health-related habits, family history, current medication information from pill bottles, previous medical diagnoses, etc.) were recorded according to standard protocols. The assessment and definitions of lifestyle factors, hypertension and diabetes are detailed in the Online Appendix.

### Physical examination and laboratory measurements

The detailed methods used for physical examinations and laboratory evaluations have been previously described (11). Briefly, body weight, height, waist circumference (WC), heart rate and blood pressure were measured. BMI was calculated as weight in kilograms divided by height in meters squared (kg/m^2^).

After the participants fasted overnight for at least 8 h, blood samples were collected and immediately processed and analyzed for alanine aminotransferase (ALT), aspartate aminotransferase (AST), albumin, total bilirubin, uric acid, creatinine, glucose, lipids and platelet count at the clinical laboratory of the Third Xiangya Hospital. The sample analysis was performed in accordance with the manufacturer’s specifications. FIB-4 was used as a noninvasive and accurate marker of fibrosis based on the following formula: FIB-4 index = age × AST(U/L)/platelet count (×10^9^/L) $\surd \mathrm{ALT}\left(U/L\right)$ (12). The estimated glomerular filtration rate (eGFR) was used as an index of renal disease based on the modification of diet in renal disease formula for Chinese subjects: eGFR = 175 × Scr^−1.234^ × age^−0.179^ [if female, ×0.79] (13).

### Determination of NAFLD and clinical phenotypes

NAFLD was defined as the presence of hepatic steatosis without excessive alcohol consumption (≥30 g/d in men and ≥20 g/d in women) or concomitant liver disease (14). The method used for detecting hepatic steatosis was hepatic ultrasound (Logiq 9, GE Medical System, Milwaukee, WI, United States). Positive abdominal ultrasound findings included the following 5 criteria: (1) parenchymal brightness, (2) liver-to-kidney contrast, (3) deep beam attenuation, (4) bright vessel walls and (5) gallbladder wall definition (15). Subjects with at least two abnormal findings were diagnosed with hepatic steatosis (16).

In accordance with the Working Group on Obesity in China (17), BMIs of 18.5–23.9 kg/m^2^ were defined as normal weight; BMIs of 24.0–27.9 kg/m^2^ were defined as overweight and ≥28.0 kg/m^2^ were defined as obesity. The MetS definition in the National Cholesterol Education Program-Adult Treatment Panel III International Diabetes Federation (IDF) criteria (18) included elevated triglyceride (TG) levels (≥1.69 mmol/L) or the use of lipid-lowering drugs, low high-density lipoprotein (HDL) cholesterol levels (<1.03 mmol/L in men and <1.29 mmol/L in women), elevated systolic blood pressure (≥130 mmHg) or diastolic blood pressure (≥85 mmHg) or the use of antihypertensive drugs, and elevated blood glucose levels (≥5.6 mmol/L) or the use of any medications for diabetes (insulin or oral glucose-lowering medications). WC was not included because of collinearity with BMI. Individuals with one or fewer of these components were deemed metabolically healthy (MH), with two or more being deemed metabolically unhealthy (MU) according to the Framingham Heart Study (19).

Based on the combination of BMI categories and metabolic health status, the clinical phenotypes of NAFLD were then categorized into 6 groups: normal weight-MH, normal weight-MU, overweight-MH, overweight-MU, obese-MH and obese-MU.

### Assessment of baPWV and carotid plague

As previously reported (11), baPWV was measured with an automatic waveform analyzer (BP-203 RPE III, Omron Health Medical, Dalian, China). After a minimum rest of 5 min in the supine position, 4 cuffs were wrapped around the extremities (upper arms and ankles) and then connected to the plethysmography sensor (volume pulse form) and an oscillometric pressure sensor. Pressure waveforms were recorded at both the brachial and tibial arteries to assess the transmission time between the initial increases in these waves. The measurements were performed twice, and the mean of the left- and right-side baPWV values were calculated. Moreover, substantial side differences in the baPWV of more than 10 m/s indicated problems with measurement, and the measurement should be repeated. The highest baPWV quartile (>1,590 cm/s) was classified as increased baPWV (arterial stiffness) (20).

As previously reported (21), carotid plaque was assessed using carotid artery sonography (Siemens AcusonSequoiaTM512 Ultrasound System, Mountain View, CA, United States) with a 9 MHz linear array transducer. Experienced sonographers performed carotid examinations, including bilateral visualization of the common, internal, and external carotid arteries. Carotid plaque was defined as a focal wall thickening of at least 0.5 mm or 50% of the surrounding CIMT that encroached into the arterial lumen or a focal region with CIMT greater than 1.5 mm that protruded into any carotid segment (22).

### Statistical analyses

Descriptive characteristics are presented as the mean ± SD or median (interquartile range) for continuous variables and as the number (percentage) for categorical variables. The covariates between different groups were compared using a t test or the Mann–Whitney *U* test for continuous variables and the chi-square test for categorical variables. Multivariate logistic regression models were performed to assess the association of baPWV (the highest quartile versus other quartiles) and carotid plaques (presence versus absence) with (1) the 6 clinical phenotypes above; (2) the 3 BMI categories (normal weight, overweight and obese); (3) the 4 different categories of metabolic abnormalities (0, 1, 2 and ≥3 indices); and (4) individual metabolic abnormality risk factors (elevated blood pressure, triglyceridemia, hyperglycemia levels and low HDL-cholesterol). In addition, multiple linear regression analysis was used to evaluate the associations between baPWV (defined as a continuous variable) and the 6 clinical phenotypes. Potential covariates were adjusted for age, sex, demographic characteristics, lifestyles, WC, heart rate, albumin, total bilirubin, FIB-4, uric acid and eGFR.

Additional sensitivity analysis was performed to replicate our main findings with stricter definitions of metabolically healthy status, including the WC criterion (cutoff points of ≥90 cm for men and ≥85 cm for women) for the definition and defining metabolically healthy participants as having none of the five possible metabolic abnormality risk factors.

A *p*-value <0.05 was considered statistically significant. Statistical analyses were performed using SAS version 9.4 (SAS Institute, Cary, NC), and graphs were drawn by GraphPad Prism version 6.00 (GraphPad Software, La Jolla California, United States).

## Results

### Study population

The cross-sectional analysis included 27,738 overall participants (mean age: 49.8 years; 80.3% were male) with complete baPWV information in cross-sectional study sample I. A total of 14,323 individuals (mean age: 60 years; 76.7% were male) with complete carotid plaque information were included in cross-sectional study sample II.

### Prevalence and characteristics of body size phenotypes

**Figure 2:**
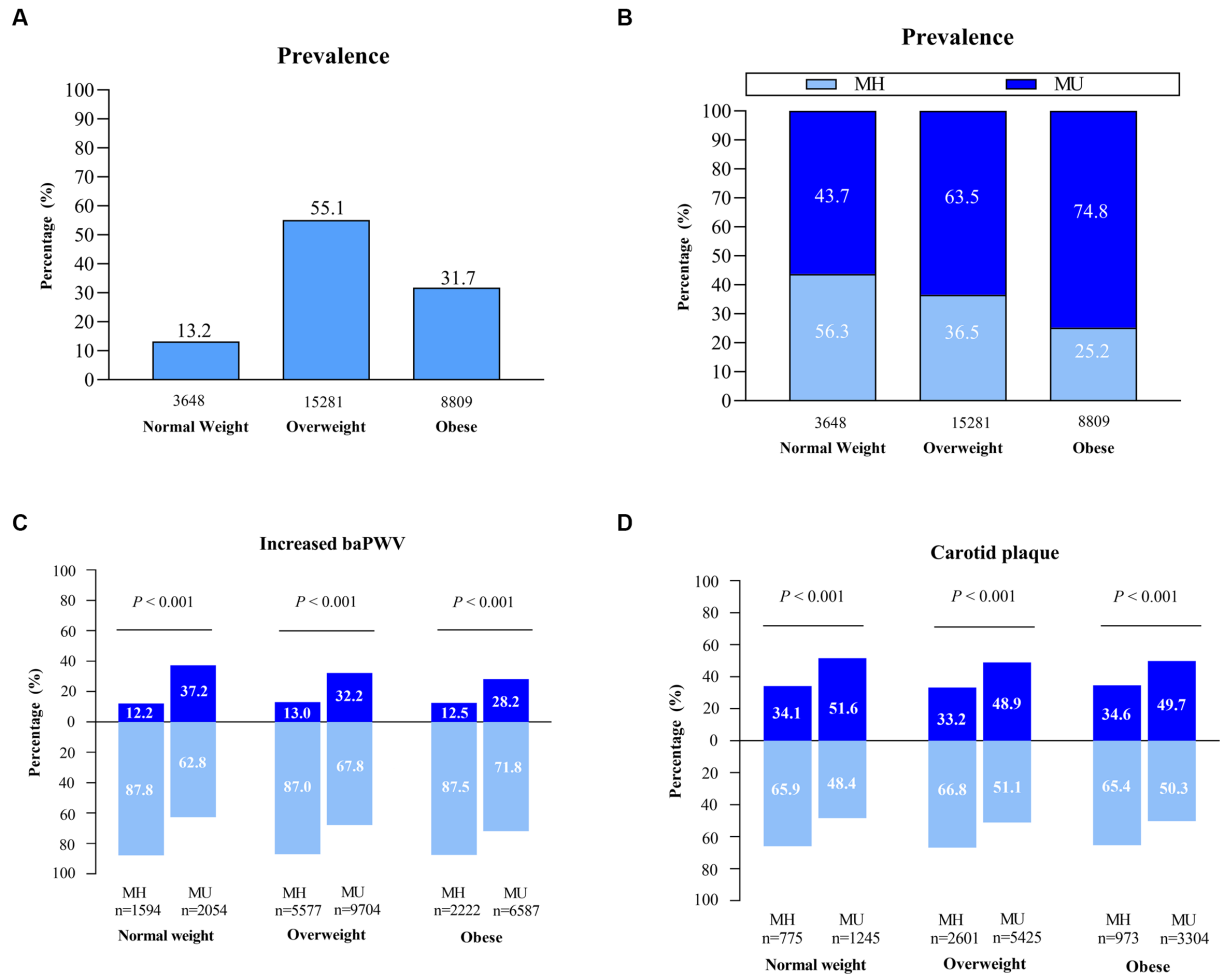
The prevalence of clinical phenotypes and distribution of subclinical atherosclerosis in the NAFLD population. **(A–C)** Assessment in NAFLD cross-sectional sample I (*n* = 27,738). **(D)** Assessment in NAFLD cross-sectional sample II (*n* = 14,323). MH, metabolically healthy; MU, metabolically unhealthy.

Of the 27,738 NAFLD participants, 13.2%, 55.1% and 31.7% fell into the normal weight, overweight and obese categories, respectively (Figure 2A). In the normal weight group, the MH subjects accounted for a higher percentage than did the MU subjects (56.3% vs. 43.7%), whereas MU status was predominant in the overweight and obese groups (63.5% vs. 36.5%; 74.8% vs. 25.2%) (Figure 2B).

Within each BMI strata, MU individuals were older, had a higher proportion of poor diet scores, had a higher prevalence of diabetes and hypertension, and had higher WC, heart rate, blood pressure, and levels of fasting glucose, triglycerides, liver enzymes and uric acid than MH individuals. In contrast, a greater proportion of MH participants had a university degree, a worker occupation and a short sleep duration, and they had significantly greater levels of HDL-C and total bilirubin than participants with MU (Table 1).

**Table 1:** Study population clinical characteristics stratified by clinical phenotypes in cross-sectional sample I.

	Overall	Normal weight		Overweight		Obese	
		MH	MU	MH	MU	MH	MU
Prevalence, *n* (%)	27,738 (100%)	1,594 (5.7%)	2054 (7.4%)	5,577 (20.1%)	9,704 (35.1%)	2,222 (8.0%)	6,587 (23.7%)
*Demographic factors*							
Age, years	49.8 ± 10.7	50.3 ± 10.2	53.6 ± 10.3	48.8 ± 10.6	51.3 ± 10.3	46.9 ± 11.1	48.1 ± 10.7
Male sex, *n* (%)	22,274 (80.3%)	1,141 (71.6)	1,302 (63.4)	4,597 (82.4)	7,801 (80.4)	1864 (83.9)	5,569 (84.5)^ns^
University degree, *n* (%)	11,797 (42.5)	837 (52.5)	805 (39.2)	2,760 (49.5)	4,056 (41.8)	928 (41.8)	2,411 (36.6)
Being married, *n* (%)	23,872 (86.1)	1,412 (88.6)	1782 (86.8)	4,896 (87.8)	8,411 (86.7)	1843 (82.9)	5,528 (83.9)^ns^
Workers (occupation), *n* (%)	15,997 (57.7)	1,103 (69.2)	1,198 (58.3)	3,575 (64.1)	5,500 (56.7)	1,296 (58.3)	3,325 (50.5)
*Lifestyle status*							
Current smoker, *n* (%)	9,412 (33.9)	511 (32.1)	592 (28.8)	1835 (32.9)	3,272 (33.7)^ns^	795 (35.8)	2,407 (36.5)^ns^
Current drinker, *n* (%)	12,132 (43.7)	588 (36.9)	743 (36.2)^ns^	2,424 (43.5)	4,302 (44.3)^ns^	988 (44.5)	3,087 (46.9)^ns^
*Physical activity, n (%)*							
Inactive	12,004 (43.3)	631 (39.6)	868 (42.3)^ns^	2,193 (39.3)	4,064 (41.9)	1,024 (46.1)	3,224 (48.9)^ns^
Moderately active	4,351 (15.7)	238 (14.9)	292 (14.2)^ns^	832 (14.9)	1,550 (16.0)	368 (16.6)	1,071 (16.3)^ns^
Active	11,383 (41.0)	725 (45.5)	894 (43.5)^ns^	2,552 (45.8)	4,090 (42.1)	830 (37.4)	2,292 (34.8)^ns^
*Healthy diet*							
Poor	11,126 (40.1)	500 (31.4)	724 (35.2)	1968 (35.3)	3,960 (40.8)	941 (42.3)	3,033 (46.0)
Intermediate	15,874 (57.2)	1,022 (64.1)	1,266 (61.6)	3,421 (61.3)	5,494 (56.6)	1,228 (55.3)	3,443 (52.3)
Ideal	738 (2.7)	72 (4.5)	64 (3.1)	188 (3.4)	250 (2.6)	53 (2.4)	111 (1.7)
Short sleep duration, *n* (%)	9,004 (32.5)	738 (46.3)	758 (36.9)	2065 (37.0)	3,014 (31.1)	690 (31.1)	1739 (26.4)
*Classic vascular risk factors*							
Body-mass index, kg/m^2^	26.9 ± 2.8	22.8 ± 0.97	22.9 ± 0.93 ^ns^	26.0 ± 1.1	26.1 ± 1.1	29.8 ± 1.7	30.3 ± 2.1
Waist circumference, cm	91.2 ± 7.7	81.6 ± 4.9	82.4 ± 4.9	88.8 ± 5.1	89.7 ± 5.2	97.1 ± 6.3	98.5 ± 6.6
Heart rate, beats/min	73.5 ± 11.0	71.0 ± 10.2	75.3 ± 11.3	70.6 ± 10.0	74.2 ± 11.1	71.7 ± 10.3	75.7 ± 11.2
Systolic blood pressure, mm Hg	130.9 ± 16.3	120.8 ± 13.6	132.6 ± 16.5	123.1 ± 14.1	134.1 ± 15.8	125.4 ± 14.0	136.3 ± 16.0
Diastolic blood pressure, mm Hg	81.9 ± 11.3	75.2 ± 9.3	81.1 ± 10.7	77.1 ± 9.6	84.0 ± 10.9	78.6 ± 10.1	86.2 ± 11.4
Hypertension, *n* (%)	7,021 (25.3)	142 (8.9)	532 (25.9)	708 (12.7)	2,917 (30.1)	367 (16.5)	2,355 (35.8)
Anti-hypertensive medication, *n* (%)	1,544 (5.6)	12 (0.8)	89 (4.3)	78 (1.4)	663 (6.8)	68 (3.1)	634 (9.6)
Fasting glucose, mmol/L	5.6 (5.1, 6.2)	5.2 (4.9, 5.4)	5.8 (5.3, 6.6)	5.2 (4.9, 5.5)	5.9 (5.4, 6.6)	5.2 (4.9, 5.5)	5.9 (5.4, 6.7)
Diabetes mellitus, *n* (%)	3,937 (14.2)	74 (4.6)	425 (20.7)	201 (3.6)	188 (19.4)	75 (3.4)	1,276 (19.4)
Anti-diabetes medication, *n* (%)	553 (2.0)	12 (0.8)	63 (3.1)	22 (0.4)	276 (2.8)	11 (0.5)	169 (2.6)
Triglycerides, mmol/L	2.1 (1.5, 3.1)	1.4 (1.1, 1.8)	2.3 (1.8, 3.3)	1.5 (1.1, 2.0)	2.5 (1.8, 3.6)	1.5 (1.2, 2.1)	2.6 (1.9, 3.8)
HDL-C cholesterol, mmol/L	1.22 ± 0.27	1.4 ± 0.30	1.2 ± 0.29	1.32 ± 0.25	1.18 ± 0.25	1.27 ± 0.23	1.13 ± 0.23
LDL-C cholesterol, mmol/L	2.86 ± 0.91	3.05 ± 0.81	3.07 ± 0.96^ns^	3.03 ± 0.79	3.04 ± 0.94^ns^	3.06 ± 0.77	3.06 ± 0.94^ns^
Anti-dyslipidemia medication, *n* (%)	274 (1.0)	2 (0.1)	16 (0.8)	5 (0.1)	139 (1.4)	7 (0.3)	105 (1.6)
*Emerging risk factors and others*							
ALT, U/L	31.0 (22.0, 44.0)	24.0 (18.0, 33.0)	26.0 (20.0, 37.0)	28.0 (21.0, 39.0)	31.0 (22.0, 44.0)	32.0 (23.0, 47.3)	37.0 (25.0, 54.0)
AST, U/L	21.0 (25.0, 27.0)	24.0 (20.0, 27.0)	25.0 (20.0, 27.0)	25.0 (20.0, 27.0)	25.0 (21.0, 27.0)	25.0 (21.0, 28.0)	25.0 (22.0, 30.0)
Albumin, g/L	45.3 ± 10.6	45.9 ± 7.0	45.5 ± 9.9^ns^	45.8 ± 8.3	45.3 ± 10.8^ns^	45.3 ± 10.0	44.7 ± 12.8^ns^
Total bilirubin, μmol/L	14.8 ± 5.8	16.0 ± 5.9	14.9 ± 6.2	15.6 ± 5.8	14.6 ± 5.8	14.8 ± 5.9	13.9 ± 5.3
FIB-4	0.36 (0.24, 0.52)	0.43 (0.29, 0.62)	0.43 (0.30, 0.61)^ns^	0.37 (0.26, 0.54)	0.37 (0.25, 0.54)^ns^	0.32 (0.21, 0.47)	0.31 (0.21, 0.46)^ns^
Uric acid, mmol/L	387 ± 88	346 ± 82	363 ± 88	375 ± 83	388 ± 86	393 ± 88	409 ± 89
eGFR, mL/min/1.73m^2^	102 (89, 117)	105 (91, 120)	106 (92, 122)^ns^	101 (89, 116)	101 (88, 116)^ns^	100 (89, 114)	101 (88, 117)

Data are presented as the mean ± standard deviation, median (interquartile range), or count (percentage). Uric acid was available for 27,712 participants, FIB-4 was available for 27,710 participants, and ALB and total bilirubin was available for 27,708 participants. The remaining baseline characteristics were available for all subjects. MH, metabolically healthy; MU, metabolically unhealthy; BMI, body mass index; BP, blood pressure; LDL-C, low-density lipoprotein cholesterol; HDL-C, high-density lipoprotein cholesterol; ALT, alanine aminotransferase; AST: aspartate aminotransferase; FIB-4, eGFR, estimated glomerular filtration rate. ^ns^Nonsignificant for the comparison between MH (metabolic healthy) and MU (metabolic unhealthy) within each BMI strata. The remaining comparisons were statistically significant.

### Subclinical atherosclerosis profile

The MU individuals had an obviously higher prevalence of increased baPWV and carotid plaque than the MH individuals across all BMI categories. The extent of subclinical atherosclerosis was similar among MH individuals across BMI categories. For MU individuals, the extension was relatively higher for normal-weight individuals than for overweight and obese individuals (37.2% vs. 32.2% vs. 28.2%; 51.6% vs. 48.9% vs. 49.7%) (Figures 2C,D). A possible reason is that lean NAFLD subjects tend to be older than non-lean NAFLD subjects (52.2 vs. 49.5 years).

### Association between subclinical atherosclerosis and clinical phenotypes

Increased baPWV and the presence of carotid plaque were available for the analysis in cross-sectional study sample I and sample II, respectively.

First, we evaluated the association between subclinical atherosclerosis and clinical phenotypes (6 subgroups) after adjusting for potential confounding factors (Table 2). The associations, which were defined according to the highest baPWV quartiles and carotid plaque, were evaluated using multiple logistic regression analysis. With the normal weight-MH subgroup as a reference, among MU subjects, normal weight, overweight and obesity were all significantly associated with a higher risk for increased baPWV and carotid plaque, and the adjusted odds ratio of risk was similar across BMI categories; however, among MH individuals, overweight and obesity showed a much lower but marginally significant risk of increased baPWV but were not associated with carotid plaque. Additionally, we conducted linear regression analysis for baPWV, which was defined as a continuous variable. Similar patterns were found regarding the relationship of subclinical atherosclerosis and MAFLD clinical phenotypes.

**Table 2:** The associations of clinical phenotypes with increased arterial stiffness in cross-sectional study sample I and carotid plaque in cross-sectional study sample II.

Cross-sectional sample 1 (*n* = 27,738)	Model 1		Model 2		Model 3	
	OR (95% CI)	*p*-value	OR (95% CI)	*p*-value	OR (95% CI)	*p*-value
**baPWV—binary variable** ^a^						
NAFLD normal weight-MH	Reference		Reference		Reference	
NAFLD normal weight-MU	4.09 (3.38–4.96)	<0.001	4.03 (3.32–4.89)	<0.001	3.38 (2.77–4.13)	<0.001
NAFLD overweight-MH	1.25 (1.04–1.50)	0.020	1.23 (1.02–1.48)	0.028	1.23 (1.01–1.50)	0.038
NAFLD overweight-MU	4.06 (3.42–4.82)	<0.001	3.93 (3.31–4.67)	<0.001	3.44 (2.86–4.13)	<0.001
NAFLD obese-MH	1.44 (1.16–1.78)	0.001	1.37 (1.10–1.70)	0.005	1.29 (1.01–1.64)	0.041
NAFLD obese-MU	4.51 (3.79–5.38)	<0.001	4.25 (3.56–5.07)	<0.001	3.39 (2.75–4.17)	<0.001

Cross-sectional sample 1 (*n* = 27,738)	Model 1		Model 2		Model 3	
	*β* (95% CI)	*p*-value	*β* (95% CI)	*p*-value	*β* (95% CI)	*p*-value
**baPWV—continuous variable** ^b^						
NAFLD normal weight-MH	Reference		Reference		Reference	
NAFLD normal weight-MU	159.9 (145.0–174.9)	<0.001	157.2 (142.2–172.1)	<0.001	128.9 (114.6–143.3)	<0.001
NAFLD overweight-MH	26.1 (13.4–38.8)	<0.001	24.4 (11.7–37.1)	<0.001	24.7 (12.1–37.3)	<0.001
NAFLD overweight-MU	148.7 (136.6–160.8)	<0.001	144.7 (132.6–156.8)	<0.001	122.5 (110.3–134.7)	<0.001
NAFLD obese-MH	36.3 (21.6–51.0)	<0.001	31.1 (16.3–45.8)	<0.001	22.6 (6.69–38.5)	0.005
NAFLD obese-MU	154.9 (142.4–167.4)	<0.001	147.8 (135.2–160.4)	<0.001	114.1 (99.7–128.6)	<0.001

Cross-sectional sample 2 (*n* = 14,323)	Model 1		Model 2		Model 3	
	OR (95% CI)	*p*-value	OR (95% CI)	*p*-value	OR (95% CI)	*p*-value
**carotid plaque—binary variable**						
NAFLD normal weight-MH	Reference		Reference		Reference	
NAFLD normal weight-MU	2.06 (1.70–2.49)	<0.001	1.95 (1.61–2.36)	<0.001	1.81 (1.49–2.20)	<0.001
NAFLD overweight-MH	0.96 (0.81–1.14)	0.660	0.93 (0.78–1.11)	0.429	0.89 (0.74–1.07)	0.204
NAFLD overweight-MU	1.87 (1.59–2.19)	<0.001	1.74 (1.48–2.05)	<0.001	1.57 (1.33–1.87)	<0.001
NAFLD obese-MH	1.08 (0.88–1.32)	0.466	0.99 (0.81–1.22)	0.950	0.90 (0.71–1.13)	0.346
NAFLD obese-MU	2.10 (1.78–2.49)	<0.001	1.91 (1.61–2.26)	<0.001	2.10 (1.78–2.49)	<0.001

Model 1 was adjusted for sex and age. Model 2 was further adjusted for education level, marital status, occupation, current smoking, current drinking, physical activity, sleeping duration, and diet status plus model 1. Model 3 was further adjusted for waist circumference (WC), heart rate, albumin, total bilirubin, FIB-4, uric acid and eGFR plus model 2. OR, odds ratio; MH, metabolically healthy; MU, metabolically unhealthy.

a baPWV as binary outcome for highest quartile versus the other quartiles performed by logistic regression.

b baPWV as a continuous outcome for independent variables performed by linear regression.

**Figure 3:**
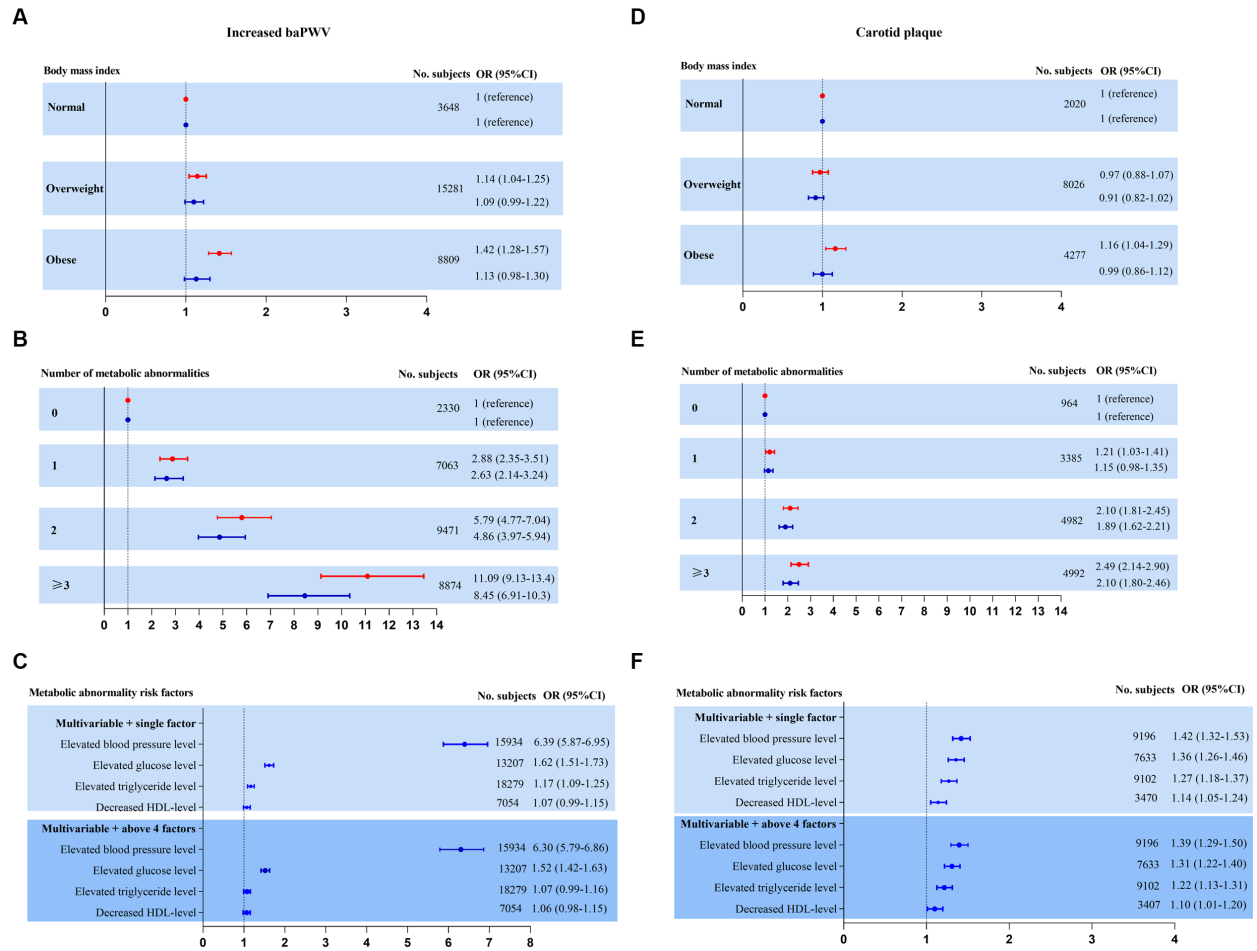
Risk for subclinical atherosclerosis stratified by body mass index, number of metabolic abnormalities and individual metabolic risk factor categories. Adjusted model 1 (red estimates) and fully adjusted model 2 (blue estimates) are both reported. Multivariate model 1 was adjusted for age and sex. Multivariate model 2 was further adjusted for education level, marital status, occupation, current smoking, current drinking, physical activity, sleeping duration, diet status, waist circumference, heart rate, ALB, total bilirubin, FIB-4, uric acid and eGFR plus model 1. **(A–C)** Increased baPWV was performed as a binary outcome for the highest quartile versus the other quartiles by logistic regression in NAFLD cross-sectional sample 1 (*n* = 27,738). **(D–F)** Carotid plaque as a binary outcome for presence versus absence by logistic regression in NAFLD cross-sectional sample 2 (*n* = 14,323).

Second, to further explore whether the increased risk was mediated by metabolic abnormalities but not by increased BMI, we performed separate analyses to further assess the association between subclinical atherosclerosis and body size phenotypes after adjustment for confounding variables (Figures 3A,B,D,E). Whereas increased baPWV and carotid plaque increased with the increase in the number of metabolic abnormalities, there was no increase in the incidence of subclinical disease across BMI categories.

Finally, the adjusted risk for subclinical disease was also calculated according to individual and joint metabolic risk factors. Both the presence of elevated blood pressure and elevated glucose levels were revealed to be obviously significant covariates in predicting subclinical disease. Elevated triglyceride levels and decreased HDL levels were borderline or not significantly associated with subclinical disease (Figures 3C,F).

### Sensitivity analyses

Using a strict definition of metabolic health led to a slight reinforcement of the magnitude of the associations. However, this did not generally affect the trend of these associations (Supplementary Table S1 and Supplementary Figure S1).

## Discussion

### Main findings

We report here, for the first time, evidence of an association between clinical phenotypes and subclinical atherosclerosis in light of two different indices. We found a wide spectrum of clinical phenotypes in the NAFLD population. The poor metabolic profile was noticeable in overweight and obese individuals. Compared with metabolically healthy subjects, metabolically unhealthy subjects had a higher burden of subclinical atherosclerosis regardless of their BMI phenotypes. Unhealthy metabolic status, but not BMI-based body size, was the main contributor to the associations of clinical phenotypes with subclinical atherosclerosis. An increasing number of comorbid MetS traits were linked with a higher risk in this cohort. Among the MetS components, elevated blood pressure and blood glucose levels were stronger risk factors associated with the prevalence of subclinical diseases. Our results held true not only when we used a clinical definition of metabolic healthy status (presence of one or fewer of the 4 metabolic risk factors) but also when we used a more stringent definition (presence of none of the 5 cardiometabolic risk factors).

### Comparison with previous studies

The presence of carotid plaque was validated as an excellent marker of atherosclerotic lesions and structural abnormalities, reflecting generalized atherosclerosis. Increased baPWV is a reliable index of early vascular functional stiffness. A growing body of studies has reported that NAFLD is associated with the two markers of preclinical atherosclerosis, notably independent of established CVD risk factors (8, 23). However, these previous studies have several limitations. First, the most frequently included populations were hospital-based or outpatient cohorts, and some patients were diagnosed with liver biopsy, which resulted in a relatively small sample and led to selection bias. Hence, the results might lead to an overestimation of the effect (24–28). Second, the subjects among whom this link was observed were simply NAFLD vs. non-NAFLD. Thus, the findings can be interpreted that the NAFLD population could be recognized as having a high risk of atherosclerotic CVD. However, it is important to determine how risk discrimination can be performed within the NAFLD population. In our study, the metabolic profile was the greatest contributor to the relationship of subclinical atherosclerosis with different clinical phenotypes. This was illustrated by the effect of risk in subclinical atherosclerosis being concentrated among subjects in the metabolic abnormality subgroups, suggesting a major role for metabolic health in comparison with BMI ranges. Our findings highlight the message that precise metabolic phenotyping assessment beyond BMI could enable proper classification of NAFLD patients.

Several previous studies have reported an association between clinical phenotypes and the prognosis of NAFLD. In *Gut*, Younes et al. (29) reported a multicenter study comparing the long-term prognosis, including the onset of diabetes and long-term CVD events, between lean and non-lean NAFLD populations. They found that hepatic and non-hepatic clinical complications of the two subgroups did not differ significantly over a decade of follow-up. In addition, they found that 77.5% of the lean NAFLD patients stayed at a normal weight at the end follow-up, which implied that the longitudinal progression to obesity did not contribute to the disease outcomes in such lean subjects. Another study (30) investigated the effect of metabolic abnormalities on the risk of hepatic prognosis among patients with NAFLD over a 9 years period. Among lean NAFLD patients with one or zero metabolic risk factors, the future risk is very low. Therefore, these studies challenged that a BMI-driven strategy for screening NAFLD subjects for cardiovascular or liver damage outcomes can be misleading and should be reevaluated as metabolic derangements.

### Potential mechanisms

Although BMI is an acceptable marker for overall adiposity, it cannot distinguish between muscle and fat and does not capture assessments of body-fat distribution, such as visceral and subcutaneous adipose tissue (31). Lean NAFLD is a common phenotype among Asian people, especially as they seem to have higher amounts of central fat deposition than White people and develop NAFLD and metabolic complications within a low BMI range (32, 33). Findings from body composition studies showed that increased BMI was a poor surrogate for increased visceral adipose tissue and intrahepatic fat, both of which are strongly associated with NAFLD and cardiometabolic risk factors (34–37). In contrast, several studies revealed that WC was a reliable anthropometric index of visceral adiposity. Additionally, it was recommended that health professionals should be trained to properly perform this simple measurement and consider it an important “vital sign” in clinical practice. Therefore, importantly, BMI might be a suboptimal parameter for evaluating the association between obesity and subclinical atherosclerosis in the NAFLD population. Further studies that include precise quantification of different fat distributions could help to resolve this issue.

In this study, MetS itself or components of metabolic syndrome were consistent correlates of subclinical atherosclerosis. A complicated and dynamic interaction between a multitude of factors, including genetic, epigenetic, nutrition and dietary factors, lifestyle factors, and gut microbiota, is likely to shape individual metabolic profiles (1). In addition, NAFLD and MetS share a bidirectional association as a cause and a consequence (38). NAFLD is accompanied by impaired insulin-mediated suppression of hepatic glucose production, leading to liver steatosis, hyperglycemia and dyslipidemia. In turn, selective hepatic insulin resistance is thought to result in the development of both hepatic and peripheral metabolic dysfunction. Several types of pathophysiological crosstalk between NAFLD and MetS, including insulin resistance, abnormal lipoprotein metabolism, chronic low-grade inflammation, lipotoxicity and excessive oxidative stress, exert a proatherogenic effect on blood vessels (7). Similarly, Lee et al. (39) and Wang et al. (40) present evidence from large cohort studies from Korea and China, respectively, on the utility of the MAFLD definition for incident CVD and the risk of all-cause deaths. As expected from the positive definition (with a set of accompanying metabolic abnormalities), it is appealing to cover more patients with CVD and death under the umbrella term MAFLD compared with NAFLD. However, a dissociation between NAFLD and insulin resistance/dyslipidemia/CVD is present. NAFLD patients with a specific “liver-genetic” background have a lower risk of CVD, which may partially explain why a subgroup of patients with NAFLD are metabolically healthy (41). In addition, NAFLD impacts metabolism and CVD risk through hepatokines, such as fetuin-A, ANGPTL3 (angiopoietin-related protein 3), FGF21 (fibroblast growth factor 21), SHBG (sex hormone-binding globulin), selenoprotein P, fetuin-B and follistatin (42).

We also tried to disentangle the effects of individual metabolic traits and found no correlation or only a weak effect of low HDL levels and hypertriglyceridemia on the risk of NAFLD atherosclerosis. Among the indices of lipid metabolism, only HDL cholesterol and triglyceride levels are used to define MetS. Nevertheless, total cholesterol and LDL cholesterol are risk factors for atherosclerotic lesions and structural abnormalities, which are not included in the definition.

### Implications

This study adds an important facet to the NAFLD cardiometabolic risk factor research spectrum in which, within the heterogeneity of body shape phenotype, metabolic health is more closely related to subclinical atherosclerosis, above and beyond adiposity defined by BMI. Our findings add to evidence that metabolic health conferred important clues to cardiometabolic risk that BMI may not explain.

In regard to the risk stratification implications, the strategy is solely stratified by BMI, and lean subjects with NAFLD may be misclassified as low risk and “metabolically healthy.” Obese NAFLD subjects who are “metabolically healthy” will be misclassified as being at higher risk. Accordingly, screening at-risk CVD populations instead of considering obesity or lean NAFLD as particular subgroups and performing a comprehensive assessment of specific metabolic profiles should be recommended. In other words, a novel definition of MH may be better suited to stratify subjects based on cardiometabolic risk parameters. In regard to the intervention management implications, lifestyle interventions remain the cornerstone of treatment. Weight loss has been shown substantial benefits not only for intrahepatic fat loss but also for improvement in metabolic parameters of glucose control and insulin sensitivity, whether NAFLD with overweight or normal weight (43). Several diet regimens were recommended, such as Mediterranean diet, low-carbohydrate diet, a low-fat diet and intermittent fasting over a regular diet (9, 44, 45). High adherence to the Mediterranean diet is associated with better profile of MetS features in NAFLD subjects owing to its close association with CVD (46). Nonetheless, further research is needed for its contribution on the development of subclinical atherosclerosis. In addition, some evidence showed that drinking three or more cups of coffee daily, dietary intake of vitamins E and C and phenolic acids had a protective association with NAFLD and its metabolic factors (47–49).

### Strengths and limitations

The strengths of our study include many NAFLD participants; data on both structural and functional markers of subclinical disease; rich covariable adjustments encompassing sociodemographic factors and related CVD risk factors; and additional sensitivity analysis with strict MetS criteria, which added robustness to our findings.

Moreover, our results need to be interpreted with certain limitations. First, our cross-sectional design fails to determine causal relationships between clinical phenotypes and the development of subclinical atherosclerosis. Second, this study population consisted mostly of men, which potentially limits the generalizability of the results. Third, although we used two different versions of the MetS definition, impaired glucose tolerance and the inflammatory index, which are important components of new metabolic dysfunction criteria, were not included in this study (50). Fourth, recent data suggest that clinical phenotypes might be transient for a large proportion of individuals (19). Thus, the presence of phenotypes during one clinical examination could not capture the natural course of the variability within individuals, which remains controversial regarding the findings. Fifth, we chose only two markers of subclinical atherosclerosis, and other surrogate markers, such as coronary artery calcification (CAC) and brachial arterial flow-mediated dilation (FMD), were not included in our analysis. Sixth, we did not include information on emerging “nontraditional” CVD risk factors, such as decreased vitamin D and adiponectin levels, which may influence the relationship (51, 52). Lastly, carotid plaque indexes (such as carotid plaque burden and maximum carotid plaque thickness) are better predictors of cardiovascular disease risk than CIMT, and we did not obtain quantitative indicators of CIMT in our study (53).

## Conclusion

In this health checkup-based study, different clinical phenotypes were associated with a greater subclinical CVD burden evaluated in subjects with NAFLD. However, this association remained significant only for the metabolic traits above and beyond the contribution of BMI-based body size. Our findings highlight the importance of metabolic status in screening fatty liver subjects at higher CVD risk. Although several treatments for NAFLD are currently in the pipeline, it would be preferable to establish treatment strategies with known benefits for improving both fat distribution and metabolic abnormalities in to achieve a better cardiovascular prognosis in the future.

## Data availability statement

The raw data supporting the conclusions of this article will be made available by the authors, without undue reservation.

## Ethics statement

The studies involving human participants were reviewed and approved by institutional review board at the Third Xiangya Hospital (No. 2018-S393). The patients/participants provided their written informed consent to participate in this study. Written informed consent was obtained from the individual(s) for the publication of any potentially identifiable images or data included in this article.

## Author contributions

YW: study design, acquisition of clinical data, statistical analysis, and writing the manuscript. TY, SD, XZ, YD, and XL: acquisition of clinical data. LL and CW: acquisition of clinical data and modification of the manuscript. All authors contributed to the article and approved the submitted version.

## Funding

This study was funded by the Changsha Natural Science Foundation (kq2208351), the National Natural Science Foundation of China (82001338), Hunan Provincial Natural Science Foundation (2020JJ4854, 2021JJ40934, and 2021JJ30989) and the Special Funding for the Construction of Innovative Provinces in Hunan (2020SK2055).

## Conflict of interest

The authors declare that the research was conducted in the absence of any commercial or financial relationships that could be construed as a potential conflict of interest.

## Publisher’s note

All claims expressed in this article are solely those of the authors and do not necessarily represent those of their affiliated organizations, or those of the publisher, the editors and the reviewers. Any product that may be evaluated in this article, or claim that may be made by its manufacturer, is not guaranteed or endorsed by the publisher.
