# Supplementary material for: Metabolic health phenotype better predicts subclinical atherosclerosis than body mass index-based obesity phenotype in the non-alcoholic fatty liver disease population
Source: Front Nutr. 2023 Sep 19;10:1104859. doi: 10.3389/fnut.2023.1104859 (PMC10546180; doi:10.3389/fnut.2023.1104859)
Supplement: Supplementary file 1 [file Data_Sheet_1.docx]

**Supplemental Materials**

**METHODS**

**Clinical characteristics**

Current smoking was coded present if more than one cigarette per day (on average) was consumed over a period longer than six months. Regular alcohol drinking was coded present if the individual reported consumption of beer, wine (including Chinese wine) and/or liquor at least two days per week over a period exceeding 12 months. Physical activity was classified as inactive, moderately active, or active according to the frequency (never, 1-2 times/week, and ≥ 3 times/week, respectively). Five components of dietary habits were evaluated according to modified diet definitions of the AHA^[1]^: 1. with cereals and legumes as the basic food; 2. ≥ 500 g (1 “Jin”) vegetables and fruits/day; 3. < 100 g (2 “Liang”) red meat/day; 4. regular (in most weeks) intake of unprocessed fish and/or soybean products; and 5. preference for non-salty food. The total score can be up to five points and categorized into three degrees as follows: ideal = 4-5 points, intermediate = 2-3 points, and poor = 0-1 point. A short sleep duration was defined as <7 h according to the American Heart Association regarding the impact of sleep duration on cardiometabolic health^[2]^.

The presence of type 2 diabetes and hypertension were defined according to the Chinese guidelines. Hypertension was defined according to the following criteria: (1) systolic BP ≥140 mmHg and/or diastolic BP ≥90 mmHg, (2) self-reported physician-diagnosed hypertension, and/or (3) antihypertensive medication. Diabetes was defined by the presence of any of the following: (1) self-reported history of physician-diagnosed diabetes; (2) current use of insulin or oral antidiabetic drugs, and/or (3) fasting plasma glucose ≥ 7.0 mmol/L.

**Physical examination and** **laboratory measurements**

Height and weight were measured on a scale, with the subjects wearing light clothing and without shoes. Waist circumference (WC) was measured from the bottom edge of the last rib and the iliac crest. The mean of the two measurements was calculated. Blood pressure (BP) was measured on the right upper arm in the sitting position after 10-15 min of rest using a validated digital automatic analyzer (Omron 9020). Systolic BP and diastolic BP were each measured twice, and the mean of the two readings was considered in the analysis.

Fasting blood glucose was measured with the glucose oxidase method. Alanine transaminase (ALT) and aspartate aminotransferase (AST) were determined by the bromocresol green (BCG) method. HDL cholesterol, LDL cholesterol, triglycerides, creatinine, and uric acid were measured with enzymatic methods. For these analyses, an automated analyzer (Hitachi 7600-110; Hitachi, Tokyo, Japan) was used. Platelet blood cells were measured using an automated cell counter (XE 5000; Sysmex, Kobe, Japan). All laboratory analyses were performed in accordance with the manufacturer's specifications.

**S Table 1.** The associations of clinical phenotypes defined by BMI and metabolic status with arterial stiffness in Cross-Sectional sample 1 and with carotid plaque in Cross-Sectional sample 2.

|  | **Model 1** | | **Model 2** | | **Model 3** | |
| --- | --- | --- | --- | --- | --- | --- |
| Cross-Sectional sample 1 (n = 27738) | OR (95%CI) | p Value | OR (95%CI) | p Value | OR (95%CI) | p Value |
| **baPWV– binary variable ^a^** |  | <0.001 |  | <0.001 |  | <0.001 |
| NAFLD normal weight–MH | Reference |  | Reference |  | Reference | Reference |
| NAFLD normal weight–MU | 6.04 (3.92–9.31) | <0.001 | 5.77 (3.74–8.89) | <0.001 | 4.44 (2.84–6.93) | < 0.001 |
| NAFLD overweight–MH | 1.04 (0.60–1.78) | 0.896 | 1.03 (0.60–1.78) | 0.906 | 1.00 (0.57–1.75) | 0.997 |
| NAFLD overweight–MU | 6.35 (4.14–9.72) | <0.001 | 5.94 (3.88–9.09) | <0.001 | 4.83 (3.11–7.49) | < 0.001 |
| NAFLD obese–MH | 1.20 (0.15–9.94) | 0.864 | 1.13 (0.14–9.43) | 0.911 | 0.97 (0.11–8.79) | 0.981 |
| NAFLD obese–MU | 7.51 (4.90–11.5) | <0.001 | 6.78 (4.42–10.4) | <0.001 | 5.12 (3.29–7.96) | < 0.001 |
| Cross-Sectional sample 1 (n = 27738) | β (95%CI) | p Value | β (95%CI) | p Value | β (95%CI) | p Value |
| **baPWV– continuous variable ^b^** |  |  |  |  |  |  |
| NAFLD normal weight–MH | Reference |  | Reference |  | Reference |  |
| NAFLD normal weight–MU | 156.4 (133.9–178.9) | <0.001 | 151.2 (128.7–173.6) | <0.001 | 116.9 (95.4–138.5) | <0.001 |
| NAFLD overweight–MH | 12.2 (-14.8–39.2) | 0.378 | 12.1 (-14.8–39.0) | 0.378 | 14.7 (-11.0–40.4) | 0.262 |
| NAFLD overweight–MU | 157.9 (136.5–179.3) | <0.001 | 150.5 (129.2–171.8) | <0.001 | 122.6 (102.2–143.1) | <0.001 |
| NAFLD obese–MH | 18.4 (-69.2–106.0) | 0.681 | 14.4 (-72.9–101.8) | 0.746 | 9.93 (-73.1–93.0) | 0.815 |
| NAFLD obese–MU | 173.5 (151.9–195.1) | <0.001 | 162.0 (140.4–183.6) | < 0.001 | 122.8 (102.0–143.6) | <0.001 |
| Cross-Sectional sample 2 (n = 14323) | OR (95%CI) | p Value | OR (95%CI) | p Value | OR (95%CI) | p Value |
| **carotid plaque– binary variable ^a^** |  | <0.001 |  | <0.001 |  | <0.001 |
| NAFLD normal weight–MH | Reference |  | Reference |  | Reference |  |
| NAFLD normal weight–MU | 2.00 (1.44–2.79) | <0.001 | 1.92 (1.38–2.68) | <0.001 | 1.68 (1.21–2.35) | 0.002 |
| NAFLD overweight–MH | 0.93 (0.61–1.40) | 0.714 | 0.93 (0.62–1.41) | 0.748 | 0.89 (0.60–1.36) | 0.605 |
| NAFLD overweight–MU | 1.87 (1.36–2.60) | <0.001 | 1.74 (1.26–2.40) | <0.001 | 1.50 (1.09–2.07) | 0.014 |
| NAFLD obese–MH | 0.53 (0.06–4.79) | 0.571 | 0.54 (0.06–5.05) | 0.587 | 0.47 (0.05–4.52) | 0.515 |
| NAFLD obese–MU | 2.19 (1.59–3.02) | <0.001 | 1.96 (1.42–2.71) | <0.001 | 2.10 (1.78–2.49) | 0.004 |

Model 1 was adjusted for sex and age.

Model 2 was further adjusted for education level, marital status, occupation, current smoking, current drinking, physical activity, sleeping duration, and diet status plus model 1.

Model 3 was further adjusted for WC, heart rate, ALB, total bilirubin, FIB–4, uric acid and eGFR plus model 2.

^a^ baPWV as binary outcome for highest quartile versus the other quartiles performed by logistic regression;

^b^ baPWV as a continuous outcome for independent variables performed by linear regression.

Abbreviations: OR, odds ratio; MH, metabolically healthy; MU, metabolically unhealthy.


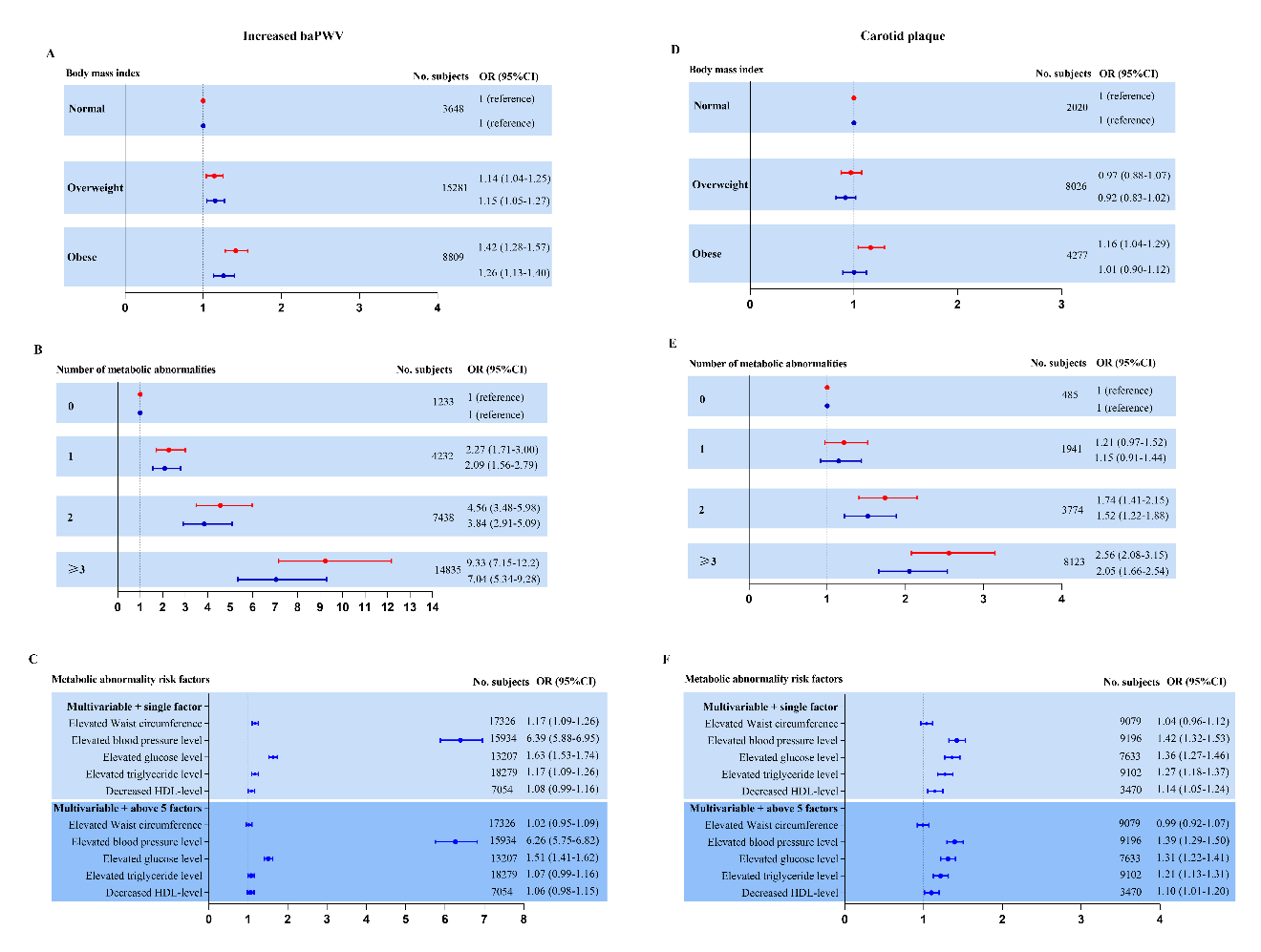


**S Figure 1.** Risk for subclinical atherosclerosis stratified by body mass index, number of metabolic abnormalities and individual metabolic risk factor categories with stricter definitions of the metabolically healthy status in sensitivity analysis. Unadjusted (red estimates) and adjusted (blue estimates) results are both reported. baPWV as a binary outcome for the highest quartile versus the other quartiles performed by logistic regression. Multivariate models were adjusted for age, sex, education level, marital status, occupation, current smoking, current drinking, physical activity, sleeping duration, diet status, heart rate, albumin, total bilirubin, FIB–4, uric acid and eGFR.

**References**

[1] Y.Q. Wang, C.F. Wang, L. Zhu, et al. Ideal cardiovascular health and the subclinical impairments of cardiovascular diseases: a cross-sectional study in central south China[J]. BMC Cardiovasc. Disord. 2017, 17(1), 269. DOI: 10.1186/s12872-017-0697-9.

[2] M.P. St-Onge, M.A. Grandner, D. Brown, et al. Sleep Duration and Quality: Impact on Lifestyle Behaviors and Cardiometabolic Health: A Scientific Statement From the American Heart Association[J]. Circulation. 2016, 134(18), e367-e386. DOI: 10.1161/CIR.0000000000000444.
